# Effectiveness of regular reporting of spirometric results combined with a smoking cessation advice by a primary care physician on smoking quit rate in adult smokers: a randomized controlled trial. ESPIROTAB study

**DOI:** 10.1186/1471-2296-12-61

**Published:** 2011-06-28

**Authors:** Mar Rodriguez-Alvarez, Pere Torán-Monserrat, Laura Muñoz-Ortiz, Antonio Negrete-Palma, Juan José Montero-Alia, Mercedes Jiménez-González, Elena Zurilla-Leonarte, Victoria Marina-Ortega, Montserrat Olle-Borque, Esther Valentin-Moya, Anna Cortada-Cabrera, Alexis Tena-Domingo, Silvia Martínez-González, Victoria Vila-Palau, Adriana Ramos-Ordoñez, Guida Rotllant-Estelrich, Carme Forcada-Vega, Eulàlia Borrell-Thió

**Affiliations:** 1 Primary Healthcare Centre Canet de Mar, Catalan Health Institute. Plaça Universitat 1, 08640 Canet de Mar (Barcelona), Spain; 2 Primary Healthcare Research Support Unit Metropolitana Nord, Catalan Health Institute-IDIAP Jordi Gol. Camí del Mig 36, 08303 Mataró (Barcelona), Spain; 3 Primary Healthcare Centre Gatassa, Catalan Health Institute. Camí del Mig 36 (4a planta), 08303 Mataró (Barcelona), Spain; 4 Primary Healthcare Centre Rocafonda-Palau, Catalan Health Institute. Ronda Pintor Rafael Estrany 24, 08304 Mataró (Barcelona), Spain; 5 Primary Healthcare Centre La Riera, Catalan Health Institute. La Riera 7, 08302 Mataró (Barcelona), Spain; 6 Primary Healthcare Centre LLefià, Catalan Health Institute. Carretera Antiga de València s/n, 08913 Badalona (Barcelona), Spain; 7 Primary Healthcare Centre Pineda de Mar, Catalan Health Institute. Carrer de Tarragona 49, 08397 Pineda de Mar (Barcelona), Spain; 8 Primary Healthcare Service Mataró-Maresme, Catalan Health Institute. Carrer Verge de Guadalupe 2, 08303 Mataró (Barcelona), Spain; 9 Primary Healthcare Centre Sant Roc, Catalan Health Institute. Carrer Vélez Rubio s/n, 08913 Badalona (Barcelona), Spain

## Abstract

**Background:**

Undiagnosed airflow limitation is common in the general population and is associated with impaired health and functional status. Smoking is the most important risk factor for this condition. Although primary care practitioners see most adult smokers, few currently have spirometers or regularly order spirometry tests in these patients. Brief medical advice has shown to be effective in modifying smoking habits in a large number of smokers but only a small proportion remain abstinent after one year. The aim of this study is to evaluate the effectiveness of regular reporting of spirometric results combined with a smoking cessation advice by a primary care physician on smoking quit rate in adult smokers.

**Methods/design:**

Intervention study with a randomized two arms in 5 primary care centres. A total of 485 smokers over the age of 18 years consulting their primary care physician will be recruited.

On the selection visit all participants will undergo a spirometry, peak expiratory flow rate, test of smoking dependence, test of motivation for giving up smoking and a questionnaire on socio-demographic data. Thereafter an appointment will be made to give the participants brief structured advice to give up smoking combined with a detailed discussion on the results of the spirometry. After this, the patients will be randomised and given appointment for follow up visits at 3, 6, 12 and 24 months. Both arms will receive brief structured advice and a detailed discussion of the spirometry results at visit 0. The control group will only be given brief structured advice about giving up smoking on the follow up. Cessation of smoking will be tested with the carbon monoxide test.

**Discussion:**

Early identification of functional pulmonary abnormalities in asymptomatic patients or in those with little respiratory symptomatology may provide "ideal educational opportunities". These opportunities may increase the success of efforts to give up smoking and may improve the opportunities of other preventive actions to minimise patient risk. Comparing adult smokers in the intervention group with those in the control group, a minimum improvement expected with respect to the rates of smoking cessation would represent a large number of avoided morbimortality.

**Trial Registration:**

ClinicalTrials.gov: NCT01296295

## Background

Chronic obstructive pulmonary disease (COPD) involves a heterogeneous group of processes characterised by little reversible air flow obstruction of chronic and progressively disabling evolution which causes a high morbimortality around the world. According to the World Health Organisation (WHO) 210 million people around the world have COPD [1] and despite being a potentially avoidable disease, has foreseen that in 2030 COPD will be the cause of 7.8% of all deaths and 27% of the deaths related to smoking only by cancer and cardiovascular diseases [2].

In addition to increasing the mortality, its prevalence is also slowly rising so epidemiological predictions foresee a growing trend in the next decades [3]. These and other reasons have converted this health care problem into a priority in the most important international institutions [4]. In our setting the high prevalence of COPD in parallel with the smoking habit have been consistently emphasised [5]. The IBERPOC study has evaluated COPD among 9% of the Spanish population between 40 to 69 years of age, a percentage that increases up to 23% in subjects over the age of 60 years [6]. At the time of the study, 48% of the population reported some respiratory symptom (13.5% cough, 10.7% chronic expectoration and 10.4% dyspnea) always higher frequency in men.

The diagnosis of COPD is clear and its treatment is feasible. However, a high number of patients remain undiagnosed and therefore not treated. Interestingly, the letters of COPD coincide with "Confusion Over Patient Diagnosis" [7]. In Spain, data of the IBERPOC study confirm that underdiagnosis is frequent (78.1%) and is accompanied by undertreatment (80.7%) [8]. The reasons include not only certain nihilistic attitudes towards treatment but also an initial symptomatology makes diagnosis difficult [9]. The preliminary results of the EPI-SCAN study performed 10 years after the IBERPOC have confirmed the high rate of underdiagnosis of COPD in Spain with a prevalence of 10.2% in the population from 40 to 80 years of age. Underdiagnosis 10 years later was slightly reduced from 78% in the IBERPOC study to 73% in EPI-SCAN. Moreover, undertreatment was reduced from 81% to 54% [10].

From a more economic point of view, the morbimortality associated with COPD has an enormous social and individual impact. It has been estimated that COPD consumes 2% of the Spanish health care budget representing approximately 0.25% of the Gross National Product. A recent study has shown an average annual cost per patient of $ 1,760 [11]. Family practitioners are in the best position to change this scenario.

Aetiopathogenics of COPD are multifactorial. A history of smoking is invariably found in most cases, although 15% to 20% of smokers develop the disease [12]. Thus, the presence of smoking habit and suggestive symptomatology make functional respiratory evaluation compulsary for early detection of respiratory disease obligatory [13]. Forced spirometry is a simple reproducible test and an objective method to quantify airflow obstruction in the early phases. Several studies have reported that forced spirometry in subjects at risk is a highly effective strategy for diagnosis of COPD. Investigators from the University of Maastricht performed this approach in the primary care setting and demonstrated its efficacy [14]. According to this study, the probability of detecting spirometric alterations in smokers over 40 years of age with chronic cough is 2.5-fold greater than in non smokers. Likewise, a multicentre study carried out in 12 primary care centres showed a prevalence of COPD of 30.6% among smokers of 10 packs/year over the age of 40 [15].

The risks derived from smoking are well known in the biomedical community and political class, and increasingly more, by the society in general. The international GOLD (Global Initiative for Chronic Obstructive Lung Disease) consensus insists that smoking is the most important risk factor for the development of COPD and thus, antismoking advice should be the first therapeutic recommendation [16]. However, this obvious objective is not at all easy to achieve. At present, 29% of the European Union population is considered to be a regular smoker. According to the last Health Care Plan of Catalonia, in 1998 the smoking prevalence in the population from 15 to 64 years of age was 37.5% (30.7% in women and 44.4% in men)[17]. The last National Health Survey of 2006 carried out by the Spanish Ministry of Health showed the smoking prevalence was 26.4% over 16 years of age (31.6% in men and 21.5% in women ) with 42% smoking ≥ 20 cigarettes / day (50.2% men and 31.6% women) [18].

Cessation of smoking is the simplest and most profitable preventive method to avoid the development of COPD. It is also the most effective therapeutic intervention in already diagnosed patients and is the only effective method to increase survival [19]. Giving up smoking has an immediate effect on both pulmonary function and the risk of hospitalisation and total mortality [20]. Many studies have shown that smoking cessation produces an improvement in respiratory symptoms and slows the decline in FEV1 in people with COPD [21].

The IBERPOC study reported that almost 70% of the subjects with mild COPD were smokers and many were in the precontemplative phase [22]. Another added problem is relapse of smoking which is of 70% during the first year in the general smoker population. These values are greater in the COPD group [23]. This fight has become a priority in the public health care setting for most governments of developed countries. As early as1986, the WHO agreed that smoking, in any of its forms, was incompatible with its strategic objectives.

Many strategies have been considered to fight smoking and these may essentially be classified into 3 large groups: market regulation, taxation on the price of tobacco, and health protection [24]. The promotion of healthy lifestyles is the most effective and efficient strategy within the public health setting. According to the evidence available, both isolated medical advice as well as therapy with pharmacologic measures, have shown to be effective in the approach towards smoking. The health care costs of treatment, such as a minimum medical intervention or using nicotine substitutes or other drugs, would be lower than resources devoted to other prevalent diseases such as arterial hypertension or hypercholesterolemia. With regard to isolated medical advice, this has shown to be effective in modifying the smoking habit in a large number of smokers, although only a small proportion of these patients remained abstinent after one year [25]. Although these values may vary according to the different studies, the differences found are small and range from a modest 2% described in the review by Law and Tang [26] to 11% achieved by Torrecilla et al [27] in an autochthonous study. According to recently published data, reprinting of air flow obstruction by spirometry together with antismoking advice would increase the percentage of success by 16.5%, compared to using pharmacologic agents [28]. The results obtained may seem to be modest but from a populational point of view any strategy to reduce the consumption of tobacco, regardless of being small in relative terms, is very beneficial in absolute numbers with respect to both avoidable diseases and cost saving in the health system.

The different strategies used to reduce smoking consumption have been evaluated by the Cochrane Library. Biomedical risk as an aid in smoking cessation was assessed and it was concluded that there are few tests on the effects of most of the biomedical tests available to evaluate risks [29]. Spirometry combined with interpretation of the results in relation to pulmonary age presented a significant effect of 13.6% on the rate of smoking cessation in the intervention group at 12 months and of 6.4% in the control group [30].

Indeed, although adequate tools for aiding patients to give up smoking are currently available, the percentages of success may be insufficient if the objective is to achieve a reduction in the prevalence of smoking. It is essential to investigate broad new populational approaches which reduce the magnitude of the problem, particularly when COPD is already present. If it is taken into account that spirometry is a simple and accessible test and that up to 70% of the smokers consult primary care physicians at least once a year, it is logical to assume that the opportunities of the family practitioners to diagnose the disease and offer antismoking advice are high and even more so when the work setting is the ideal place to do this.

## Methods/Design

### Study Objectives

#### Main objective

To evaluate the effectiveness of regular reporting of spirometric results combined with a smoking cessation advice by a primary care physician on smoking quit rate in adult smokers.

#### Secondary objective

To evaluate the validity of the peak expiratory flow rate to detect COPD in adult smokers and determine the prevalence of COPD and the severity of the limitation of airflow in adult smokers attended in primary care.

### Study design and setting

Interventional study with two randomized arms in 5 primary care centers of two health areas in the Maresme region (Catalonia, Spain). A total of 392 smokers over the age of 18 years consulting their primery care physician will be recruited.

### Study subjects

#### Study population

All the adult smoker population over the age of 18 consulting their primary care physician for any reason and who do not fulfil the exclusion criteria.

#### Exclusion criteria

Previous diagnosis of COPD by spirometry. Patients with counterindication to spirometry. Patients without a telephone. Patients with communication difficulties: cognitive and/or sensorial deterioration, language.

Patients with severe disease of poor prognosis (life expectancy less than one year). Patients with another respiratory disease: asthma, neoplasm of the respiratory tract, pulmonary thromboembolism, pulmonary tuberculosis, interstitial diseases. Patients who do not give written informed consent to participate in the study.

#### Sample size and selection method

To carry out the main objective of the study, sample size calculation will be performed according to the following parameters: detection of a difference greater than or equal to 10% between the two groups in regard to smoking cessation, a proportion of 5% of smoking cessation in one of the groups accepting an alpha risk of 0.05 and a beta risk of 0.10 in a bilateral contrast, 187 smokers are required in the control group and 187 smokers in the intervention group. It is assumed that 5% of smokers will withdraw the study.

### Study variables

The study data will be collected using a structured questionnaire and data collection sheets specifically designed for this objective. The structured questionnaire will include the following data: sociodemographics (age, sex, etc.), clinical history, smoking habit, respiratory symptomatology, smoking dependence test and smoking cessation motivation test. The data collection sheets will include information related to the spirometric results, peak expiratory flow rate and the carbon monoxide test when performed.

The following variables will be collected on forced spirometry, reference values using the ATS reference equations will be used:

- Forced vital capacity (FVC): a result ≥ 80% of the reference value will be considered normal.

- Forced expiratory volume in the first second (FEV1): a result ≥ 80% of the reference value will be considered normal.

- FEV1/FVC Index: a results ≥ 70 of the absolute values will be considered normal.

Spirometry defined by FEV1/FVC < 70% will be considered as forced spirometry with an obstructive pattern. A restrictive pattern will be that with FEV1/FVC values ≥ 70% and an FVC <80% and a mixed pattern will be defined with spirometry values of FEV1/FVC <70% and FVC <80%.

- Forced expiratory flow between 25% and 75% or mean expiratory flows (FEF 25-75%): normal results will be considered as > 60% of the reference value.

- Bronchodilator test (BDT): will be considered positive when the FEV1 after the BDT increases a percentage ≥ 12% and ≥ 200 ml in absolute values.

The following tests will also be carried out:

- Pulsioxymetry.

- Anthropometric variables: height and weight.

The following data will be collected using a questionnaire and will be compared with revision of the clinical histories in each of the phases:

- Usual symptomatology: expectoration, cough, presence of wheezing, dyspnoea.

- Sociodemographic variables: age, gender, occupation, level of education, municipality of residence and site of home (rural, semi-urban, urban).

- Exposure to tobacco (measurement in packs / year), years smoking, age at initiation of smoking, current smoking intake (measurement in cigarettes / day).

- History of disease and active medication.

### Procedures

During the selection visit the primary care physician will inform the candidates for participation as to the nature of the study and will offer them the possibility to participate in the study. If the patient agrees, informed consent will be requested, and a series of tests will be indicated (spirometry, peak expiratory flow rate, smoking dependence test, smoking cessation motivation test and a structured questionnaire on sociodemographics data, history of disease, smoking habit, respiratory symptomatology, etc).

On visit 0, the primary care physician will give a brief structured smoking cessation advice to all patients combined with a detailed and structured discussion of the spirometric results.

After this visit the patients will be randomised in two arms (control and intervention groups). Randomisation will be performed using a computer programme. Randomisation will be carried out by the Coordinating Centre.

Both groups will be followed up by telephone at three (visit 1) and six months (visit 2) and at one-year (visit 3) and two-year(visit 4). During the follow up visits brief structured smoking cessation advice will be reinforced in the control group but the spirometric results will not be discussed. In the intervention group brief structured smoking cessation advice will be reinforced with a detailed structured reminder discussion of the results obtained from the spyrometry of visit 0. Follow up visits 1 and 2 will be undertaken by telephone and the patients will be asked about their smoking habit and will be given a brief questionnaire.

One month before visits 3 and 4 both groups will undergo the same series of tests performed prior to visit 0 with the exception of the peak expiratory flow rate. Spirometry will be done only in the intervention group.

On visits 3 and 4, the same intervention will be done. On both visits 3 and 4 all the participants who report smoking withdrawal will undergo the carbon monoxide test.

### Analysis plan

To achieve the first objective the rates of withdrawd and reduction of the smoking habit of the two groups will be compared at 6, 12, and 24 months.

To achieve the second objective the sensitivity, specificity and positive and negative predictive values of peak flow compared to spirometry will be determined.

For the third objective, the proportion of COPD and the severity of air flow limitation will be determined according to different study variables (age, sex, smoking habit, symptoms...).

### Ethical considerations

The participants will be informed of the objectives of the study and the activities related to their participation in the study: number of visits, complementary tests, information of the results. The informed consent will describe the ethical conditions and the participant's right to intimacy, anonymity, confidentiality, withdrawal and information. Patients in whom a previously undiagnosed disease is detected will be provided with the usual diagnostic and therapeutic methods available through their reference physician who will be responsible for integrating the information and providing the best therapeutic and diagnostic options to the patient.

The data will be included in a database for data management with programmes of statistical analyses in which no reference will be made as to the identity of the subjects. Data confidentiality and anonymity will be ensured according to Law 15/1999 on data confidentiality during both the execution of the project and in the presentations and publications derived therewith. The investigators are committed to respecting the norms of good clinical practice, as well as the requirements of the Helsinki Declaration.

Authorisation will be requested from the directors of all the primary care centres and participating departments to access the clinical histories. The rules of the Catalan Health Institute with respect to access to clinical information for investigation will be fully respected.

This study has been approved by the Ethical Committee of Clinical Investigation of the Primary Care Research Institute Jordi Gol (Barcelona, Spain) on May 26^th ^2010.

## Discussion

The results of this study will provide better knowledge on how the results obtained with spirometry influence smokers in relation to the rate of withdrawd and aspects little known such as the use of the spirometry results to reinforce repeated and reminder brief structured antismoking advice.

The use of spirometry as a motivational tool to cease smoking currently continues to be very controversial. In 2007, Wilt et al [31] published a systematic review of the role of spirometry to increase the rates of smoking cessation and concluded that the current evidence available is insufficient to determine the benefit obtained on rates of smoking cessation with the use of spirometry compared with other cessation methods.

Of all the studies reviewed only one clinical trial evaluated the benefits of the use of spirometry with negative results. Later in 2008, Parkes et al [30] published a study in which a benefit was found in the reduction of smoking using the pulmonary age achieved in the spirometry versus the real age as the intervention. The rate of smoking abandonment achieved at 12 months was 13.6% in the intervention group versus 6.4% in the control group, with a difference of 7.2% with a confidence interval of [2.2-12.1].

Previous observational studies such as that by Gorecka et al [28] observed a benefit in the rate of smoking cessation using the results obtained in spirometry, particularly in patients with chronic airflow obstruction.

On the other hand, we expect the rate of participation in the present study to be high, based on the experience reported in previous studies, especially taking into acocunt that it is the primary physician of the patient who offers the possibility of participating and that participation does not imply invasive tests or pharmacological interventions.

With regard to the validity and reliability of the data collected, the difficulty and variability of some of the tests requested in the study for both the health care personnel as well as the patient (i.e. performing spirometry) are of note. Thus, consensus meetings among the professionals performing the study tests will be held with the aim of homogenising criteria and their practice. The SEPAR recommendations for performing forced spirometry will be followed. Likewise, meetings with the primary care physicians are foreseen to standardise the methodology of the interventions proposed in both groups of patients. In regard to the brief structured advice, the intervention recommended by the Smoking Group of the Spanish Society of Family and Community Medicine will be used.

Lastly, to reduce variability due to the measurement tools, it has been proposed that all the spirometers, peak flows and carbon monoxide tests used in the study be the same model and trademark.

In conclusion, we believe that the results of this study will provide better knowledge of the usefulness of spiromery in the increase in the currently controversial rate of smoking cessation and the utility of repeated use of brief structured advice in increasing and maintaining these rates over time.

## List of abbreviations

ATS: American Thoracic Society; BDT: Bronchodilator test; COPD: Chronic Obstructive Pulmonary Disease; CRF: Case report form; EPI-SCAN: The epidemiologic study of COPD in Spain; FEV_1_: Forced Expiratory Volume in the first second; FVC: Forced Vital Capacity; GOLD: Global Initiative for Chronic Obstructive Lung Disease; IBERPOC: Epidemiological study of chronic obstructive pulmonary disease in Spain; ICD: International Classification of Diseases; MRC: Medical Research Council; RR: Relative Risk; SEPAR: Sociedad Española de Neumología y Cirugía Torácica (Spanish Society of Pneumology and Chest Surgery); WHO: World Health Organization.

## Competing interests

The authors declare that they have no competing interests.

## Authors' contributions

MR and PT contributed to the original research idea about the Effectiveness of regular reporting of spirometric results combined with a smoking cessation advice by a primary care physician on smoking quit rate in adult smokers in primary health care centres. MR, PT, JJM, MJ, EZ, CF participated in the design of the study. LM will participate in the statistical analyses and has taken part in the design of the research protocol. AN, VM, MO, EV, AC, AT, SM, VV, AR, GR I EB will contribute to the coordination of the study and will provide technical support for the spirometric tests. All the authors will read, revise and approve the final manuscript.

## Pre-publication history

The pre-publication history for this paper can be accessed here:

http://www.biomedcentral.com/1471-2296/12/61/prepub

## ESPIROTAB study group members

They are also members of the ESPIROTAB study group:

From Primary Healthcare Canet de Mar: Isabel Martin, Carmen Gines, Brigida Pizarro, Alfonso Castilla.

From Primary Healthcare Vilassar de Dalt: Maria Rivodigo, Santiago Canut, Jaume Sanahuja.

From Primary Healthcare Premia de Mar: Laura Nieto.
